# Supplementary material for: Mechanistic insight into the role of AUXIN RESISTANCE4 in trafficking of AUXIN1 and LIKE AUX1-2
Source: Plant Physiol. 2023 Sep 30;194(1):422–33. doi: 10.1093/plphys/kiad506 (PMC10756756; doi:10.1093/plphys/kiad506)
Supplement: kiad506_Supplementary_Data [file kiad506_supplementary_data.zip › Suppemental Dataset S1/5445e4f6eaa2385e-AtAXR4/aligs/d1f0na_.56.alig.html]

Phyre 2 alignment of AtAXR4\_\_\_ with d1f0na\_


|  |  |
| --- | --- |
| Return to main results | Retrieve Phyre Job Id |


|  |  |  |  |
| --- | --- | --- | --- |
| Job Description | AtAXR4\_\_\_ | | |
| Confidence | 99.47% | Date | Tue Jan 10 14:09:39 GMT 2023 |
| Rank | 56 | Aligned Residues | 249 |
| % Identity | 18% | Template | d1f0na\_ |


| SCOP info | alpha/beta-Hydrolases | alpha/beta-Hydrolases | Mycobacterial antigens |
| Resolution | 1.80 | | |

Show / Hide SS confidence

Show / Hide Conservation and Alignment quality

|  |  |  |  |  |  |  |  |  |  |
| --- | --- | --- | --- | --- | --- | --- | --- | --- | --- |
|  | Insertion relative to template ||  | Deletion relative to template |
|  | Catalytic residue from the CSA |
|  | |
| Detailed help on interpreting your alignment | |

  
  

|  |  |  |  |  |  |  |  |  |  |  |  |  |  |  |  |  |  |  |  |  |  |  |  |  |  |  |  |  |  |  |  |  |  |  |  |  |  |  |  |  |  |  |  |  |  |  |  |  |  |  |  |  |  |  |  |  |  |  |  |  |  |
| --- | --- | --- | --- | --- | --- | --- | --- | --- | --- | --- | --- | --- | --- | --- | --- | --- | --- | --- | --- | --- | --- | --- | --- | --- | --- | --- | --- | --- | --- | --- | --- | --- | --- | --- | --- | --- | --- | --- | --- | --- | --- | --- | --- | --- | --- | --- | --- | --- | --- | --- | --- | --- | --- | --- | --- | --- | --- | --- | --- | --- | --- |
|  |  | 90 | . | . | . | . | . | . | . | . | . | 100 | . | . | . | . | . | . | . | . | . | 110 | . | . | . | . | . | . | . | . | . | 120 | . | . | . | . | . | . | . | . | . |  |  | 130 | . | . | . | . | . |  |  |  | . | . | . | . | 140 | . | . | . | . |
| Predicted Secondary structure |  | --- | --- |  |  |  |  |  |  | --- | --- | --- | --- | --- | --- | --- |  |  |  |  |  |  |  | --- | --- | --- | --- | --- | --- | --- |  |  |  |  |  | --- | --- | --- | --- | --- | --- | . | . |  |  |  |  |  |  | . | . | . |  |  |  |  |  |  |  | --- | --- |
| Query SS confidence |  | --- | --- | --- | --- | --- | --- | --- | --- | --- | --- | --- | --- | --- | --- | --- | --- | --- | --- | --- | --- | --- | --- | --- | --- | --- | --- | --- | --- | --- | --- | --- | --- | --- | --- | --- | --- | --- | --- | --- | --- | . | . | --- | --- | --- | --- | --- | --- | . | . | . | --- | --- | --- | --- | --- | --- | --- | --- | --- |
| Query Sequence |  | N | G | R | T | I | K | V | Q | V | N | S | N | E | S | P | I | E | V | F | V | A | E | S | G | S | I | H | T | E | T | V | V | I | V | H | G | L | G | L | S | . | . | S | F | A | F | K | E | . | . | . | M | I | Q | S | L | G | S | K | G |
| Query Conservation |  |  |  |  |  |  |  |  |  |  |  |  |  |  |  |  | --- |  |  |  |  |  | --- |  | --- |  |  |  |  |  | --- | --- | --- | --- | --- | --- | --- |  |  |  | --- | . | . | --- |  |  | --- | --- |  | . | . | . | --- |  |  |  | --- | --- |  |  | --- |
| Template Conservation |  |  | --- | --- |  |  |  | --- |  | --- |  |  |  |  |  |  | --- |  |  |  | --- |  |  | --- |  |  |  |  | . | --- | --- | --- |  |  | --- | --- | --- |  |  | --- |  |  |  |  |  |  |  |  |  |  |  |  |  |  |  |  |  |  |  |  |  |
| Template Sequence |  | P | V | E | Y | L | Q | V | P | S | P | S | M | G | R | D | I | K | V | Q | F | Q | S | G | G | N | N | S | . | P | A | V | Y | L | L | D | G | L | R | A | Q | D | D | Y | N | G | W | D | I | N | T | P | A | F | E | W | Y | Y | Q | S | G |
| Template Known Secondary structure |  | --- |  |  |  |  |  |  |  |  | T | T | T | T |  |  |  |  |  |  |  |  | --- | --- | --- | T | T | --- | . | --- |  |  |  |  |  | --- | --- | T | T | --- | --- | S | S | S | --- |  |  |  |  |  | S | --- |  |  |  |  |  | T | T | S | S |
| Template Predicted Secondary structure |  |  |  |  |  |  |  |  |  | --- | --- | --- | --- | --- | --- |  |  |  |  |  |  |  | --- | --- | --- | --- | --- | --- | . |  |  |  |  |  |  | --- | --- | --- | --- | --- | --- | --- | --- | --- |  |  |  |  |  |  | --- | --- |  |  |  |  |  |  | --- | --- | --- |
| Template SS confidence |  | --- | --- | --- | --- | --- | --- | --- | --- | --- | --- | --- | --- | --- | --- | --- | --- | --- | --- | --- | --- | --- | --- | --- | --- | --- | --- | --- | --- | --- | --- | --- | --- | --- | --- | --- | --- | --- | --- | --- | --- | --- | --- | --- | --- | --- | --- | --- | --- | --- | --- | --- | --- | --- | --- | --- | --- | --- | --- | --- | --- |
|  |  | 7 | . | . | 10 | . | . | . | . | . | . | . | . | . | 20 | . | . | . | . | . | . | . | . | . | 30 | . | . | . |  | . | . | . | . | . | . | 40 | . | . | . | . | . | . | . | . | . | 50 | . | . | . | . | . | . | . | . | . | 60 | . | . | . | . | . |
|  |  |  |  |  |  |  |  |  |  |  |  |  |  |  |  |  |  |  |  |  |  |  |  |  |  |  |  |  |  |  |  |  |  |  |  |  |  |  |  |  |  |  |  |  |  |  |  |  |  |  |  |  |  |  |  |  |  |  |  |  |  |
|  |  | 145 | . | . | . | . | 150 | . | . | . | . | . | . | . | . | . | 160 | . | . | . | . | . | . | . | . | . | 170 | . | . | . | . | . | . | . | . | . | 180 | . | . | . | . | . | . | . | . | . | 190 | . | . | . | . | . | . | . | . | . | 200 | . | . | . | . |
| Predicted Secondary structure |  | --- |  |  |  |  |  | --- | --- | --- | --- | --- | --- | --- | --- | --- | --- | --- | --- | --- | --- | --- | --- | --- | --- | --- | --- | --- | --- | --- | --- | --- | --- | --- | --- |  |  |  | --- | --- | --- | --- | --- | --- | --- | --- | --- | --- | --- | --- | --- | --- | --- | --- | --- | --- | --- | --- | --- | --- | --- |
| Query SS confidence |  | --- | --- | --- | --- | --- | --- | --- | --- | --- | --- | --- | --- | --- | --- | --- | --- | --- | --- | --- | --- | --- | --- | --- | --- | --- | --- | --- | --- | --- | --- | --- | --- | --- | --- | --- | --- | --- | --- | --- | --- | --- | --- | --- | --- | --- | --- | --- | --- | --- | --- | --- | --- | --- | --- | --- | --- | --- | --- | --- | --- |
| Query Sequence |  | I | H | S | V | A | I | D | L | P | G | N | G | F | S | D | K | S | M | V | V | I | G | G | D | R | E | I | G | F | V | A | R | V | K | E | V | Y | G | L | I | Q | E | K | G | V | F | W | A | F | D | Q | M | I | E | T | G | D | L | P | Y |
| Query Conservation |  |  | --- | --- | --- | --- |  | --- | --- | --- | --- |  | --- |  | --- |  |  | --- |  |  |  |  |  |  |  |  |  |  |  |  |  |  |  |  |  |  |  |  |  |  |  |  |  |  |  |  |  |  |  |  |  |  |  |  |  |  |  |  |  |  |  |
| Template Conservation |  |  |  | --- | --- |  |  |  |  |  |  |  |  |  |  |  |  |  |  |  |  |  |  |  |  |  |  |  |  |  |  |  |  | . | . | . | . | . | . | . | . | . | . | . | . | . | . | . | . | . | . | . | . | . | . | . | . | . | . | . | . |
| Template Sequence |  | L | S | I | V | M | P | V | G | G | Q | S | S | F | Y | S | D | W | Y | S | P | A | C | G | K | A | G | C | Q | T | Y | K | W | . | . | . | . | . | . | . | . | . | . | . | . | . | . | . | . | . | . | . | . | . | . | . | . | . | . | . | . |
| Template Known Secondary structure |  | --- |  |  |  |  |  | --- | --- | --- | T | T | --- | T | T | --- | B | --- | S | S | --- |  |  |  | T | T |  |  |  | --- | --- | B |  | . | . | . | . | . | . | . | . | . | . | . | . | . | . | . | . | . | . | . | . | . | . | . | . | . | . | . | . |
| Template Predicted Secondary structure |  | --- |  |  |  |  |  |  | --- | --- | --- | --- | --- | --- | --- | --- | --- | --- | --- | --- | --- | --- | --- | --- | --- | --- | --- | --- | --- | --- | --- | --- |  | . | . | . | . | . | . | . | . | . | . | . | . | . | . | . | . | . | . | . | . | . | . | . | . | . | . | . | . |
| Template SS confidence |  | --- | --- | --- | --- | --- | --- | --- | --- | --- | --- | --- | --- | --- | --- | --- | --- | --- | --- | --- | --- | --- | --- | --- | --- | --- | --- | --- | --- | --- | --- | --- | --- | --- | --- | --- | --- | --- | --- | --- | --- | --- | --- | --- | --- | --- | --- | --- | --- | --- | --- | --- | --- | --- | --- | --- | --- | --- | --- | --- | --- |
|  |  | 66 | . | . | . | 70 | . | . | . | . | . | . | . | . | . | 80 | . | . | . | . | . | . | . | . | . | 90 | . | . | . | . | . | . | . |  |  |  |  |  |  |  |  |  |  |  |  |  |  |  |  |  |  |  |  |  |  |  |  |  |  |  |  |
|  |  |  |  |  |  |  |  |  |  |  |  |  |  |  |  |  |  |  |  |  |  |  |  |  |  |  |  |  |  |  |  |  |  |  |  |  |  |  |  |  |  |  |  |  |  |  |  |  |  |  |  |  |  |  |  |  |  |  |  |  |  |
|  |  | 205 | . | . | . | . | 210 | . | . | . | . | . | . | . | . | . | 220 | . | . | . | . | . | . | . | . | . | 230 | . | . | . | . | . | . | . | . | . |  |  |  | 240 | . | . | . | . | . | . | . | . | . | 250 | . | . | . | . | . | . | . | . | . | 260 | . |
| Predicted Secondary structure |  | --- | --- | --- | --- | --- | --- | --- | --- | --- | --- | --- | --- | --- | --- | --- | --- | --- | --- | --- | --- |  |  |  |  |  |  |  |  |  |  |  |  |  |  | --- | . | . | . | --- | --- | --- | --- |  |  |  |  |  |  | --- | --- |  |  |  |  |  |  |  |  |  |  |
| Query SS confidence |  | --- | --- | --- | --- | --- | --- | --- | --- | --- | --- | --- | --- | --- | --- | --- | --- | --- | --- | --- | --- | --- | --- | --- | --- | --- | --- | --- | --- | --- | --- | --- | --- | --- | --- | --- | . | . | . | --- | --- | --- | --- | --- | --- | --- | --- | --- | --- | --- | --- | --- | --- | --- | --- | --- | --- | --- | --- | --- | --- |
| Query Sequence |  | E | E | I | I | K | L | Q | N | S | K | R | R | S | F | K | A | I | E | L | G | S | E | E | T | A | R | V | L | G | Q | V | I | D | T | L | . | . | . | G | L | A | P | V | H | L | V | L | H | D | S | A | L | G | L | A | S | N | W | V | S |
| Query Conservation |  |  |  |  |  |  |  |  |  |  |  |  |  |  |  |  |  |  |  |  |  |  |  |  |  | --- |  |  | --- |  |  |  | --- | --- |  | --- | . | . | . | --- | --- |  |  |  |  | --- | --- | --- | --- | --- |  | --- | --- |  | --- | --- |  |  |  | --- |  |
| Template Conservation |  | . | . | . | . | . | . | . | . | . | . | . | . | . | . | . | . | . | . | . | . |  |  |  |  |  |  | --- | --- |  |  |  |  |  |  |  |  |  | --- | --- |  |  |  |  |  | --- |  | --- |  | --- |  | --- | --- |  |  | --- | --- |  |  | --- |  |
| Template Sequence |  | . | . | . | . | . | . | . | . | . | . | . | . | . | . | . | . | . | . | . | . | E | T | F | L | T | S | E | L | P | Q | W | L | S | A | N | R | A | V | K | P | T | G | S | A | A | I | G | L | S | M | A | G | S | S | A | M | I | L | A | A |
| Template Known Secondary structure |  | . | . | . | . | . | . | . | . | . | . | . | . | . | . | . | . | . | . | . | . |  |  |  |  |  | T |  |  |  |  |  |  |  |  |  |  | --- | --- | --- | S | S | S |  |  |  |  |  |  | T |  |  |  |  |  |  |  |  |  |  |  |
| Template Predicted Secondary structure |  | . | . | . | . | . | . | . | . | . | . | . | . | . | . | . | . | . | . | . | . |  |  |  |  |  |  |  |  |  |  |  |  |  |  |  | --- | --- | --- | --- |  |  |  |  |  |  |  |  |  | --- |  |  |  |  |  |  |  |  |  |  |  |
| Template SS confidence |  | --- | --- | --- | --- | --- | --- | --- | --- | --- | --- | --- | --- | --- | --- | --- | --- | --- | --- | --- | --- | --- | --- | --- | --- | --- | --- | --- | --- | --- | --- | --- | --- | --- | --- | --- | --- | --- | --- | --- | --- | --- | --- | --- | --- | --- | --- | --- | --- | --- | --- | --- | --- | --- | --- | --- | --- | --- | --- | --- | --- |
|  |  |  |  |  |  |  |  |  |  |  |  |  |  |  |  |  |  |  |  |  |  | 98 | . | 100 | . | . | . | . | . | . | . | . | . | 110 | . | . | . | . | . | . | . | . | . | 120 | . | . | . | . | . | . | . | . | . | 130 | . | . | . | . | . | . | . |
|  |  |  |  |  |  |  |  |  |  |  |  |  |  |  |  |  |  |  |  |  |  |  |  |  |  |  |  |  |  |  |  |  |  |  |  |  |  |  |  |  |  |  |  |  |  |  |  |  |  |  |  |  |  |  |  |  |  |  |  |  |  |
|  |  | 262 | . | . | . | . | . | . | . | 270 | . | . | . | . | . | . | . | . | . | 280 | . | . | . | . | . | . | . | . | . | 290 | . | . | . | . | . | . | . | . | . | 300 | . | . | . | . | . | . | . | . | . | 310 | . | . | . | . | . | . | . | . | . | 320 | . |
| Predicted Secondary structure |  |  | --- |  |  |  |  | --- |  |  |  |  |  | --- | --- | --- | --- | --- | --- | --- | --- |  |  |  |  |  |  | --- |  |  |  |  |  |  |  |  |  | --- | --- |  |  |  |  |  |  |  |  |  |  | --- | --- | --- | --- | --- | --- |  |  |  |  |  |  |
| Query SS confidence |  | --- | --- | --- | --- | --- | --- | --- | --- | --- | --- | --- | --- | --- | --- | --- | --- | --- | --- | --- | --- | --- | --- | --- | --- | --- | --- | --- | --- | --- | --- | --- | --- | --- | --- | --- | --- | --- | --- | --- | --- | --- | --- | --- | --- | --- | --- | --- | --- | --- | --- | --- | --- | --- | --- | --- | --- | --- | --- | --- | --- |
| Query Sequence |  | E | N | W | Q | S | V | R | S | V | T | L | I | D | S | S | I | S | P | A | L | P | L | W | V | L | N | V | P | G | I | R | E | I | L | L | A | F | S | F | G | F | E | K | L | V | S | F | R | C | S | K | E | M | T | L | S | D | I | D | A |
| Query Conservation |  |  |  | --- | --- | --- | --- |  |  | --- | --- | --- | --- | --- | --- |  |  |  |  |  |  | --- |  |  | --- |  |  |  |  |  |  |  |  |  |  |  |  |  |  |  |  |  |  |  |  | --- |  |  |  |  |  |  |  |  |  |  | --- |  | --- |  |  |
| Template Conservation |  |  |  | --- | --- |  | --- |  |  | --- |  |  |  | --- | --- |  | . | . | . | . | . | . | . | . | . | . | . | . | . | . | . | . | . | . | . | . | . | . | . | . | . | . | . | . | . | . | . |  |  |  |  |  |  |  |  |  |  |  |  |  |  |
| Template Sequence |  | Y | H | P | Q | Q | F | I | Y | A | G | S | L | S | A | L | . | . | . | . | . | . | . | . | . | . | . | . | . | . | . | . | . | . | . | . | . | . | . | . | . | . | . | . | . | . | . | L | D | P | S | Q | G | M | G | P | S | L | I | G | L |
| Template Known Secondary structure |  |  | --- | T | T | T |  |  |  |  |  |  |  | S | --- | --- | . | . | . | . | . | . | . | . | . | . | . | . | . | . | . | . | . | . | . | . | . | . | . | . | . | . | . | . | . | . | . | S | --- | T | T | S | T | T |  |  |  |  |  |  |  |
| Template Predicted Secondary structure |  |  | --- | --- | --- | --- | --- | --- |  |  |  |  |  | --- | --- | --- | . | . | . | . | . | . | . | . | . | . | . | . | . | . | . | . | . | . | . | . | . | . | . | . | . | . | . | . | . | . | . | --- | --- | --- | --- | --- | --- | --- | --- | --- |  |  |  |  |  |
| Template SS confidence |  | --- | --- | --- | --- | --- | --- | --- | --- | --- | --- | --- | --- | --- | --- | --- | --- | --- | --- | --- | --- | --- | --- | --- | --- | --- | --- | --- | --- | --- | --- | --- | --- | --- | --- | --- | --- | --- | --- | --- | --- | --- | --- | --- | --- | --- | --- | --- | --- | --- | --- | --- | --- | --- | --- | --- | --- | --- | --- | --- | --- |
|  |  | 138 | . | 140 | . | . | . | . | . | . | . | . | . | 150 | . | . |  |  |  |  |  |  |  |  |  |  |  |  |  |  |  |  |  |  |  |  |  |  |  |  |  |  |  |  |  |  |  | . | . | . | . | . | . | . | 160 | . | . | . | . | . | . |
|  |  |  |  |  |  |  |  |  |  |  |  |  |  |  |  |  |  |  |  |  |  |  |  |  |  |  |  |  |  |  |  |  |  |  |  |  |  |  |  |  |  |  |  |  |  |  |  |  |  |  |  |  |  |  |  |  |  |  |  |  |  |
|  |  | 322 | . | . | . | . | . | . | . | 330 | . | . | . | . | . | . | . | . | . | 340 | . | . | . | . | . | . | . | . | . | 350 | . | . | . | . | . | . | . | . | . | 360 | . | . | . | . | . | . | . | . | . | 370 |  |  |  |  |  |  |  |  |  |  |  |
| Predicted Secondary structure |  |  |  |  |  |  |  | --- | --- | --- | --- |  |  |  |  |  |  |  |  |  |  | --- | --- | --- | --- | --- | --- | --- |  |  |  |  |  |  |  | --- | --- | --- | --- | --- | --- |  |  |  |  |  | --- | --- | --- | --- | . | . | . | . | . | . | . | . | . | . | . |
| Query SS confidence |  | --- | --- | --- | --- | --- | --- | --- | --- | --- | --- | --- | --- | --- | --- | --- | --- | --- | --- | --- | --- | --- | --- | --- | --- | --- | --- | --- | --- | --- | --- | --- | --- | --- | --- | --- | --- | --- | --- | --- | --- | --- | --- | --- | --- | --- | --- | --- | --- | --- | . | . | . | . | . | . | . | . | . | . | . |
| Query Sequence |  | H | R | I | L | L | K | G | R | N | G | R | E | A | V | V | A | S | L | N | K | L | N | H | S | F | D | I | A | Q | W | G | N | S | D | G | I | N | G | I | P | M | Q | V | I | W | S | S | E | A | . | . | . | . | . | . | . | . | . | . | . |
| Query Conservation |  |  |  |  |  |  |  |  |  |  |  |  |  | --- |  |  |  |  |  |  |  |  |  |  |  |  |  |  |  |  |  |  |  |  | --- |  |  | --- |  | --- | --- | --- | --- | --- | --- | --- | --- |  |  | --- | . | . | . | . | . | . | . | . | . | . | . |
| Template Conservation |  |  |  |  |  |  |  |  |  |  |  |  |  |  |  |  |  |  |  |  |  |  |  |  |  |  |  |  |  |  |  |  |  |  |  |  |  |  |  |  |  |  |  |  |  |  | --- |  |  |  |  |  |  |  |  |  |  |  |  |  |  |
| Template Sequence |  | A | M | G | D | A | G | G | Y | K | A | A | D | M | W | G | P | S | S | D | P | A | W | E | R | N | D | P | T | Q | Q | I | P | K | L | V | A | N | N | T | R | L | W | V | Y | C | G | N | G | T | P | N | E | L | G | G | A | N | I | P | A |
| Template Known Secondary structure |  |  |  |  |  | T | T | S | --- | --- |  |  |  |  |  | --- | S | T | T | S |  |  |  |  |  | T | --- | T | T | T | T |  |  |  |  |  |  | T | T | --- |  |  |  |  |  | --- | --- | --- | S | --- | --- | B | T | T | B | --- | --- | S |  |  |  |
| Template Predicted Secondary structure |  |  |  |  |  |  | --- | --- | --- | --- |  |  |  | --- | --- | --- | --- | --- | --- |  |  |  |  |  |  |  |  |  |  |  |  |  |  |  | --- | --- | --- | --- | --- | --- |  |  |  |  |  |  | --- | --- | --- | --- | --- | --- | --- | --- | --- | --- | --- | --- | --- | --- | --- |
| Template SS confidence |  | --- | --- | --- | --- | --- | --- | --- | --- | --- | --- | --- | --- | --- | --- | --- | --- | --- | --- | --- | --- | --- | --- | --- | --- | --- | --- | --- | --- | --- | --- | --- | --- | --- | --- | --- | --- | --- | --- | --- | --- | --- | --- | --- | --- | --- | --- | --- | --- | --- | --- | --- | --- | --- | --- | --- | --- | --- | --- | --- | --- |
|  |  | 167 | . | . | 170 | . | . | . | . | . | . | . | . | . | 180 | . | . | . | . | . | . | . | . | . | 190 | . | . | . | . | . | . | . | . | . | 200 | . | . | . | . | . | . | . | . | . | 210 | . | . | . | . | . | . | . | . | . | 220 | . | . | . | . | . | . |
|  |  |  |  |  |  |  |  |  |  |  |  |  |  |  |  |  |  |  |  |  |  |  |  |  |  |  |  |  |  |  |  |  |  |  |  |  |  |  |  |  |  |  |  |  |  |  |  |  |  |  |  |  |  |  |  |  |  |  |  |  |  |
|  |  |  |  |  | 371 | . | . | . | . | . | . | . | . | 380 | . | . | . | . | . |  |  |  |  |  | . | . | . | . | 390 | . | . | . | . | . |  | . | . |  | . | . | 400 | . | . | . | . | . | . | . | . | . | 410 | . | . | . | . | . | . | . | . | . | 420 |
| Predicted Secondary structure |  | . | . | . | --- | --- | --- | --- |  |  |  |  |  |  |  |  |  |  | --- | . | . | . | . | . | --- | --- | --- |  |  |  |  |  | --- | --- | . | --- | --- | . | --- | --- | --- | --- | --- | --- |  |  |  |  |  |  |  |  |  |  |  |  |  |  | --- | --- | --- |
| Query SS confidence |  | . | . | . | --- | --- | --- | --- | --- | --- | --- | --- | --- | --- | --- | --- | --- | --- | --- | . | . | . | . | . | --- | --- | --- | --- | --- | --- | --- | --- | --- | --- | . | --- | --- | . | --- | --- | --- | --- | --- | --- | --- | --- | --- | --- | --- | --- | --- | --- | --- | --- | --- | --- | --- | --- | --- | --- | --- |
| Query Sequence |  | . | . | . | S | K | E | W | S | D | E | G | Q | R | V | A | K | A | L | . | . | . | . | . | P | K | A | K | F | V | T | H | S | G | . | S | R | . | W | P | Q | E | S | K | S | G | E | L | A | D | Y | I | S | E | F | V | S | L | L | P | K |
| Query Conservation |  | . | . | . |  |  | --- |  |  |  |  | --- | --- |  | --- | --- |  |  | --- | . | . | . | . | . | --- |  | --- | --- |  | --- |  | --- |  | --- | . | --- | --- | . |  | --- | --- | --- | --- |  | --- |  | --- | --- | --- |  |  | --- |  | --- | --- | --- |  |  | --- | --- |  |
| Template Conservation |  |  |  |  |  |  |  |  |  |  |  |  |  |  | --- |  |  |  | --- |  |  |  |  | --- |  |  |  |  |  |  |  |  | --- |  | --- |  | --- |  | --- |  |  | --- |  |  | . | . |  | --- |  |  |  | --- | --- |  | --- |  |  |  | --- | --- |  |
| Template Sequence |  | E | F | L | E | N | F | V | R | S | S | N | L | K | F | Q | D | A | Y | N | A | A | G | G | H | N | A | V | F | N | F | P | P | N | G | T | H | S | W | E | Y | W | G | A | . | . | Q | L | N | A | M | K | G | D | L | Q | S | S | L | G | A |
| Template Known Secondary structure |  |  |  |  |  |  |  |  |  |  |  |  |  |  |  |  |  |  |  |  |  | T | T | --- | --- | S |  |  |  |  | --- | --- | S | --- | --- | --- | S | S |  |  |  |  |  |  | . | . |  |  |  |  |  |  |  |  |  |  |  |  |  | T | --- |
| Template Predicted Secondary structure |  | --- | --- |  |  |  |  |  |  |  |  |  |  |  |  |  |  |  |  |  |  | --- | --- | --- | --- | --- |  |  |  |  |  |  | --- | --- | --- | --- | --- | --- |  |  |  |  |  |  | . | . |  |  |  |  |  |  |  |  |  |  |  |  |  | --- | --- |
| Template SS confidence |  | --- | --- | --- | --- | --- | --- | --- | --- | --- | --- | --- | --- | --- | --- | --- | --- | --- | --- | --- | --- | --- | --- | --- | --- | --- | --- | --- | --- | --- | --- | --- | --- | --- | --- | --- | --- | --- | --- | --- | --- | --- | --- | --- | --- | --- | --- | --- | --- | --- | --- | --- | --- | --- | --- | --- | --- | --- | --- | --- | --- |
|  |  | 227 | . | . | 230 | . | . | . | . | . | . | . | . | . | 240 | . | . | . | . | . | . | . | . | . | 250 | . | . | . | . | . | . | . | . | . | 260 | . | . | . | . | . | . | . | . | . |  |  | 270 | . | . | . | . | . | . | . | . | . | 280 | . | . | . | . |
|  |  |  |  |  |  |  |  |  |  |  |  |  |  |  |  |  |  |  |  |  |  |  |  |  |  |  |  |  |  |  |  |  |  |  |  |  |  |  |  |  |  |  |  |  |  |  |  |  |  |  |  |  |  |  |  |  |  |  |  |  |  |

|  |  |  |
| --- | --- | --- |
| Download: | Text version | FASTA version |

No model constructed - rank, confidence too low

  

  

---

Phyre is now FREE for commercial users!

All images and data generated by Phyre2 are free to use in any
publication with acknowledgement

Accessibility Statement

**Please cite:** The Phyre2 web portal for protein modeling, prediction and analysis

Kelley LA *et al.* *Nature Protocols*
10, 845-858 (2015)
[paper]
[Citation
link]

|  |  |
| --- | --- |
| © Structural Bioinformatics Group, Imperial College, London |  |
| Lawrence Kelley, Michael Sternberg |  |
| Disclaimer |  |
| Terms and Conditions |  |

Phyre2 is part of **Genome3D**
